# DNA Barcode Sequencing from Old Type Specimens as a Tool in Taxonomy: A Case Study in the Diverse Genus *Eois* (Lepidoptera: Geometridae)

**DOI:** 10.1371/journal.pone.0049710

**Published:** 2012-11-21

**Authors:** Patrick Strutzenberger, Gunnar Brehm, Konrad Fiedler

**Affiliations:** 1 Department of Tropical Ecology and Animal Biodiversity, University of Vienna, Vienna, Austria; 2 Institut für Spezielle Zoologie und Evolutionsbiologie mit Phyletischem Museum, Friedrich-Schiller-Universität, Jena, Germany; Natural History Museum of Denmark, University of Copenhagen, Denmark

## Abstract

In this study we report on the sequencing of the COI barcode region from 96 historical specimens (92 type specimens +4 non-types) of *Eois*. *Eois* is a diverse clade of tropical geometrid moths and is the target of a number of ongoing studies on life-histories, phylogeny, co-evolution with host plants or parasitoids, and diversity patterns across temporal and spatial dimensions. The unequivocal application of valid names is crucial for all aspects of biodiversity research as well as monitoring and conservation efforts. The availability of barcodes from historical type specimens has the potential to facilitate the much-needed acceleration of species description. We performed non-destructive DNA extraction on the abdomens of *Eois* specimens between 79 and 157 years of age. We used six primer combinations (recovering between 109 and 130 bp each) to target the full-length barcode sequence of each specimen. We were able to obtain sequences for 91 of 96 specimens (success rate 94.8%). Sequence length ranged from 121 bp to full barcode sequences (658 bp), the average sequence length was ∼500 bp. We detected a moderately strong and statistically significant negative correlation between specimen age and total sequence length, which is in agreement with expectations. The abdomen proved to be an exceedingly valuable source of DNA in old specimens of Lepidoptera. Barcode sequences obtained in this study are currently being used in an effort towards a step-wise taxonomic revision of *Eois*. We encourage that DNA barcodes obtained from types specimens should be included in all species descriptions and revisions whenever feasible.

## Introduction

The moth genus *Eois* is a significant part of megadiverse assemblages of herbivorous insects in tropical montane forest habitats in Ecuador. *Eois* accounts for up to 8.1% of all geometrid species and up to 10.2% of all geometrid individuals at certain localities [1], [2]. Local species diversity of *Eois* was explored by DNA barcoding [3]. The use of DNA barcodes in an integrative taxonomy approach lead to an increase of the species count from one single small region by up to 61% depending on the applied species concept. 94% of these taxa could not be unambiguously assigned to described species based on their external morphology. At least 145 species from southern Ecuador now await taxonomic description. *Eois* was in the past and continues to be the target of a number of studies on life-histories [4], [5], phylogeny [6] and molecular dating [7]. A comprehensive taxonomic revision of the genus and descriptions of these many newly discovered species are therefore urgently needed. This will serve to establish stable names for basic research and for long-term biomonitoring. In *Eois*, like in many tropical arthropod groups, most named species were described decades ago, with a peak at the end of the 19^th^ and beginning of the 20^th^ century [8]. At that time morphology-based species descriptions tended to be brief and vague, which exacerbates the challenge of matching sampled specimens with existing names. In contrast, substantial campaigns to unravel diversity of such tropical arthropods by means of quantitative community-wide samples [1], [2] or clarification of trophic relationships [4], [5] have largely been confined to the last two decades. Evaluation of such new samples makes it necessary to assess which of the observed specimens belong to already described species and which represent hitherto unknown lineages.

In the course of these studies it was revealed that most species names within *Eois* were incorrectly applied in the past (P. Strutzenberger, personal observation). The proper and reliable application of existing species names is crucial for interfacing and integration of data between research groups working on the same group of organisms. In the case of Neotropical *Eois* three research groups have collected substantial life-history datasets in massive efforts over the past years [4], [9], [10]. The currently very unreliable application of names, or in many cases the complete lack of names in data repositories, severely limits the value of these ecological datasets. For example, understanding the ecology and evolution of larval host-plant affiliations [4], [5], [6] or parasitoid associations [5], [11] within *Eois* requires that researchers apply the same names to the same entities of organisms. Establishing the identity of described species is also central to any effort to describe new species or any revisional work. DNA barcoding [12] can serve as a valuable source of data to achieve that goal. In contrast to most morphological methods (e.g. genitalic morphology) it provides for inexpensive, rapid and in many cases unequivocal assignment of specimens for specialists and non-specialists alike [12]. Cost and time are critical factors in biodiversity research especially when investigators are faced with mega-diverse taxa as in the case of *Eois*. DNA sequences and DNA barcodes in particular can be incorporated into taxonomy in a number of ways. DNA barcodes are routinely being used to screen for unknown species in incompletely known communities (e.g. [13], [14]). Sequences obtained from historic type specimens will probably see their main use as an additional data source for the description of new species and taxonomic revisions. This additional source of taxonomic information will be particularly valuable for otherwise poorly known taxa. Procedures for the incorporation of genetic data into taxonomy have been outlined by [15], [16]. The availability of barcodes and other sequences obtained from type specimens, therefore, has the potential to increase both the accuracy and the speed of the taxonomic process.

However, amplification of DNA from historical specimens is faced with a number of potential problems. DNA degrades over time [17], [18]. Accordingly, DNA fragment length rapidly decreases within the first few years and continues to decline at a slower rate afterwards. [17] found that average fragment length remains fairly stable in 20 to 60 years old specimens. However, the range of fragment sizes does continually narrow down. No fragments long enough to amplify the entire COI barcode region in one piece remain after a certain point in time. [18] investigated the kinetics and modes of DNA degradation with next generation sequencing data obtained from much older fossil DNA. Data on fragment size for specimens in the 80–160 year age range is currently scarce. [19] reported average fragment lengths of 87.5 and 67 bp respectively for dry (107 years old) and ethanol preserved (119 years old) specimens of the extinct thylacine. In addition to DNA degradation with time the improper treatment of specimens during collecting or preparation, e.g. treatment with formalin [17] or keeping specimens in relaxing jars for extended periods of time [20], can cause problems with PCR amplification. Unfavourable storage conditions may also be a major constraint on DNA quality. For example, the commonly used pesticide dichlorvos has detrimental effect on amplification success after only a few months of exposure [21]. Special precautions have to be taken during laboratory processing of old specimens to avoid contaminations and therefore ensure the authenticity of the obtained sequences [22]. A number of studies reported on the sequencing of DNA from dried old insect specimens. [23] successfully amplified partial COI and 28S sequences from up to 50-year-old beetles. [24] even obtained partial COI sequences from up to 192-year-old beetles. Both [23], [24] used whole specimen extraction protocols that are unsuitable for Lepidoptera due to damage done to the wing scales when submerged in a liquid. Therefore, alternative extraction protocols need to be established for Lepidoptera. A few studies exist that dealt specifically with old specimens of Lepidoptera. [25] achieved high sequencing success (94.5%) in 53–97 year old (average age ∼67 years) specimens. [26], [27] used sequences from a single or a few 75 to 150 years old lepidopteran type specimens to resolve taxonomic problems. Compared to our investigation, all of the studies mentioned above were either focused on considerably younger specimens or addressed a much smaller number of samples. The present study is the first to report on the sequencing of a large sample of old type specimens in the 79 to 157 year age range. In this study we report on technical aspects of the sequencing of DNA barcodes from old lepidopteran specimens as well as the potential for application of these sequences in taxonomy and biodiversity science.

## Materials and Methods

DNA was extracted from abdomens of 96 *Eois* specimens. 92 of these specimens are type specimens (59 holotypes, 32 syntypes (designated as lectotypes by G. Brehm), 1 paralectotype). Non-types were substituted for types whenever the type specimen was either lost or its abdomen was missing. All specimens are housed in the Natural History Museum London, UK. Specimens ranged in age from 79–157 years at the time of sequencing. For 48 specimens where the actual collection year has not been explicitly documented we deducted the average time (4 years) between collection and description of all species with known collection years. No details on how the specimens were treated immediately after collecting are preserved. It can be assumed that all specimens have been stored in similar conditions since they have been collected. All material has been in London for the last 40 years (Britsh Museum of Natural History, later Natural History Museum), some of it for longer. Most specimens were part of the Rothschild collection at Tring and moved to London in 1972. See Table S1 for a detailed list of specimens and Genbank accession numbers.

### DNA Extraction

Abdomens were digested following a protocol adapted from [20]. Abdomens were first removed from the specimen with forceps. All instruments were soaked in a sodium hypochlorite solution (12% free chlorine) for at least two minutes between handling of each specimen. According to [28] a sodium hypochlorite solution containing 0.5% free chlorine renders DNA undetectable by EtBr staining within one minute. Any residual sodium hypochlorite was removed by washing and wiping with ethanol. Abdomens were then immersed in 180 µl buffer ATL (Qiagen DNEasy Blood and Tissue Kit) and incubated at 56°C for 30 min. 10 µl Proteinase K was added and the specimen incubated for a further 90 min. Subsequently, 20 µl Proteinase K was added and the specimen digested over night at 56°C. The digest was then aspirated with a pipette and transferred to a new tube. Genitalic slides were prepared immediately after digestion. This procedure for digestion of abdomens can be considered non-destructive in the sense that no additional damage compared to conventional preparation of genitalia slides is inflicted on the specimen. If desired the abdomens can be stored in a freezer for dissection at a later point in time. Digestion of specimens was performed at the Natural History Museum London in a laboratory that is commonly used for the preparation of genitalic slides and other anatomical preparations. No PCR amplification or other DNA work is performed in this laboratory. Eppendorf filter tips were used during the entire process. Digests were stored in London for approx. 6 months at −20°C, transported to Vienna in a refrigerated container and stored at −20°C for approx. 3 months until DNA extraction. DNA extracts were repatriated to the NHM London for long-term storage after sequencing had been completed.

DNA was extracted from digests according to the standard protocol supplied with the Qiagen DNEasy Blood and Tissue Kit. DNA was eluted in 100 µl elution buffer. DNA extraction was performed in the laboratory of the Department of Tropical Ecology and Animal Biodiversity, Vienna, Austria. All pre-PCR steps were segregated from post-PCR steps by performing them in dedicated rooms or dedicated UV sterilized workstations. Surfaces were decontaminated with bleach (12% free chlorine) for 15 min immediately prior to work. Dedicated reagents were used for all pre-PCR steps. PCR reactions were performed in batches of 16 specimens. Specimens were arranged into batches to maximize morphological divergence between specimens in order to facilitate the detection of cross-contamination.

### PCR and Sequencing

PCR reactions were set up as follows: 2.5 µl of 10× (NH_4_)_2_SO_4_ PCR buffer, 2 µl MgCl_2_ (25 mM/l), 0.1 µl dNTPs (10 mM/µl), 1 µl of each primer (10 pg/µl), 1 µl template DNA, 1 µl Taq polymerase and filled to 25 µl with PCR-grade H_2_O. A PCR cycler program modified from [12] was used. PCR success was checked on a gel. PCR reactions were sequenced even if no product could be detected on a gel. PCR reactions were purified by digestion with shrimp alkaline phosphatase and exonuclease for 15 min at 37°C followed by 15 min at 80°C for enzyme deactivation. Sequencing reactions were set up with 1 µl ABI BigDye 3.1 (Applied Biosystems, Carlsbad, CA, USA), 1 µl primer, 1 µl template DNA and filled to 10 µl with PCR grade H_2_O and sequenced on an ABI capillary sequencer. PCR products were sequenced in both directions. Sequencing was performed at an in-house facility. PCR and sequencing primers for the barcode region of the mitochondrial cytochrome oxidase I (COI) gene were adapted from [27], [29]. A consensus sequence of all *Eois* reference barcode sequences available at the time (561 sequences) [3,G. Brehm, unpublished data] was generated and used as guide for primer design. Modifications to primers were done manually. Empirical tests on fresh material were performed prior to the sequencing of type specimens. It was determined that for three out of six regions the modified primers performed better whereas the original primers [27],[29] performed better on the other three regions. Primers were used accordingly (Table 1). Modified primers are designated with the prefix ‘Eois’ whereas unmodified primers are designated with the prefix ‘Sph’. Contig assembly was performed with DNAStar Lasergene version 8. Sequences were aligned manually using BioEdit [30]. Sequences were deposited into Genbank, accessions numbers JQ424345-JQ424435, for accession numbers for individual specimens see Table S1.

**Table 1:** PCR and sequencing primers: List of PCR and sequencing primers with the expected length of each amplicon indicated.

Name	Sequence	Amplicon length	Reference
LepF	5′-ATT CAA CCA ATC ATA AAG ATA TTG G-3′		Hebert 2003
COI_bc_EoisR1	5′-GCN CAY GCC TTC ATT ATA ATT TTC-3′	130 bp	this study
			
COI_bc_EoisF2	5′-CCT GGA TCT YTA ATT GGI GAT GA-3′		this study
COI_bc_EoisR2	5′-GCT TTY CCI CGA ATA AAT AAT A-3′	124 bp	this study
			
COI_bc_SphF3	5′-GGA TTT GGT AAT TGA CTA RTT CC-3′		Rougerie
COI_bc_SphR3	5′-GGA TGA ACA GTA TAA CCA CCY YT-3′	121 bp	Rougerie
COI_bc_SphF4	5′-AGT ATT GTA GAA AAT GGA GCT GG-3′		Rougerie
COI_bc_SphR4	5′-GGA GCH ATT AAY TTT ATT ACA AC-3′	109 bp	Rougerie
			
COI_bc_SphF5	5′-ATT TTT TCC CTT CAT TTR GCT GG-3′		Rougerie
COI_bc_SphR5	5′-CCA GTA TTA GCA GGA GCA ATT AC-3′	136 bp	Rougerie
			
COI_bc_EoisF6	5′-TTT GTA TGA GCT GTI GGA ATY ACT GC-3′		this study
LepR	5′-TGA TTT TTT GGA CAT CCA GAA GTT TA-3′	130 bp	Hebert 2003

### Sequence Verification and Analysis

Sequences were examined for stop codons with MEGA 5.05 for MacOSX [31] to control for numt amplification. The probability of recovering authentic mtDNA is also likely to increase due to nuclear DNA being less likely to be successfully amplified in old samples [32], [33]. Sequences were checked for contamination by construction of a neighbour-joining tree (Figure S1) with the *Eois* reference barcode dataset (804 sequences) [3,G. Brehm & P. Strutzenberger, unpublished data]. Each specimen’s position in the tree was then checked for concordance with its external morphology. We acknowledge that cross contamination between closely related species of *Eois* may go unnoticed. However, specimens were processed in batches that maximize morphological divergence among the specimens, which makes the occurrence of such errors very unlikely. The NJ-tree was also used to estimate the implications these sequences will have on *Eois* taxonomy. We applied a 2% pairwise sequence divergence threshold to approximate species boundaries. Delimitation of species with a threshold of 2% has proven to be the most useful threshold for *Eois* [3] as well as other groups of Lepidoptera [34], [35]. Logistic regressions were calculated with Statistica 8.0 to assess whether the likelihood of obtaining partial sequences with each of the six primers depended on voucher age. A linear regression between voucher age and total obtained sequence length has been calculated with Microsoft Excel.

## Results and Discussion

### Sequencing Success

**Figure 1:**
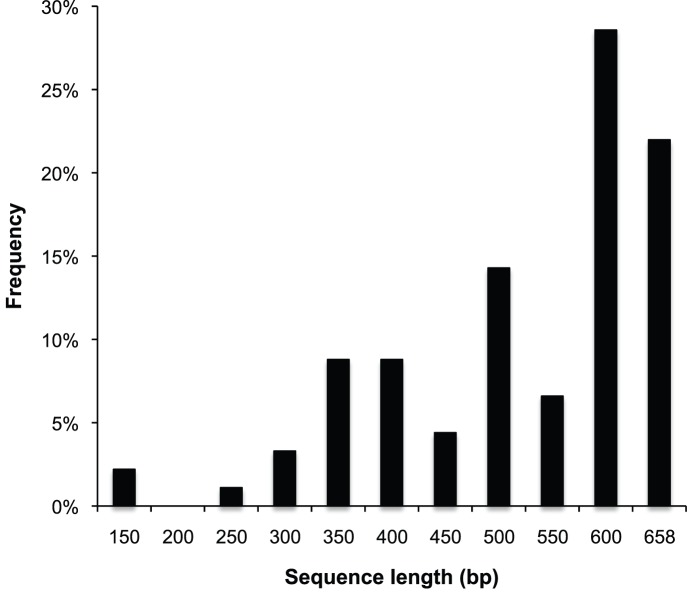
Sequence length distribution. Chart showing the frequency distribution of total sequence lengths obtained from type specimens of Neotropical *Eois* moths.

Assessment of an arbitrarily selected subset of DNA extracts on an agarose gel revealed that ∼80% of extracts contained easily detectable amounts of DNA. DNA fragment size, estimated by comparison with a DNA length marker, was approximately 80–180 bp. Empirical tests on a subset of specimens with the primer combination LepF/LepR, amplifying the entire 658 bp barcode region [12], did not result in any successful PCR amplifications. This is not surprising when considering the short fragment lengths observed in the DNA extracts. We were able to obtain sequences for 91 out of 96 processed samples. Five specimens failed to amplify for any of the six primer combinations. PCR success was virtually identical to sequencing success. There were only three cases where a PCR product could not be successfully sequenced. Repeated freeze and thaw cycles of the tissue digests or DNA during the preparation and transport of the samples did not seem to have a noticeable negative effect on PCR success. Assembled sequences ranged in length from 121 to 658 bp with a mean length of 496 bp and a median length of 557 bp. Distribution of sequence lengths (Fig. 1) shows that 57% of all specimens yielded sequences longer than 500 bp. No evidence of cross contamination could be detected. In ten cases there is indication that type specimens are likely conspecific (<2% pairwise distance) with a species present in the reference dataset from recent field samples. Type specimens did cluster with closely related species (>2% pairwise divergence) in 60 additional cases. In these latter cases the operational taxonomic units (OTUs) recognized by [1], [3] are likely not conspecific with any type specimens. In 21 cases where no closely related *Eois* species was present in the reference dataset the type sequences were not closely associated with any other sequences. No stop codons were detected in any of the obtained sequences.

**Figure 2:**
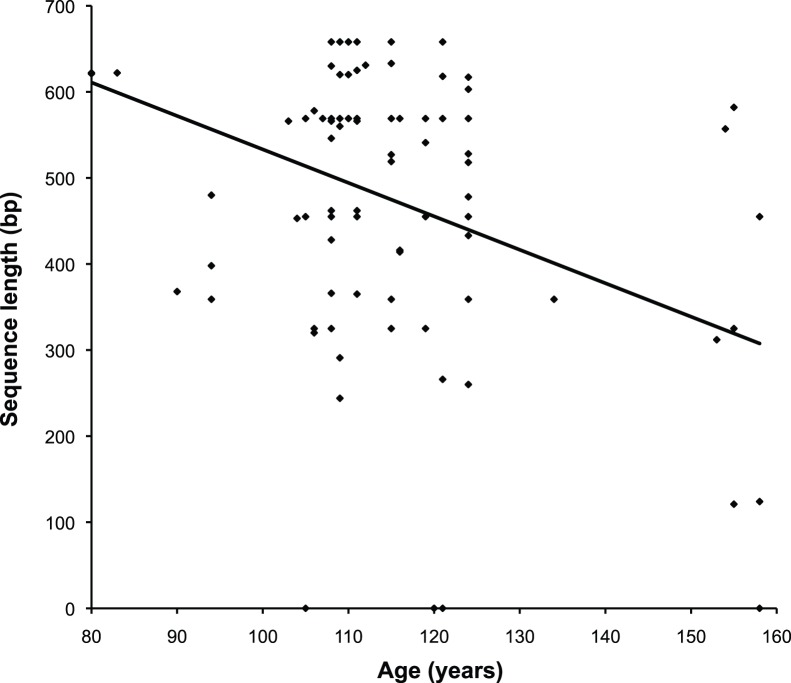
Sequence length vs. specimen age: Scatterplot of specimen age versus total obtained sequence length. A linear regression line has been fitted to the data; R^2^ = 0.148, p<0.001.

Primer combinations Eois1, Eois2, Sph3 and Eois6 returned satisfactory success rates of 83.3%, 91.7%, 81.3% and 79.2%, respectively. Primer pairs Sph4 and Sph5 had unsatisfactory low success rates of 34.4% and 60.4%, respectively. High amplification success for primer combinations Eois1, Eois2, Sph3 and Eois6 indicates that equal success is in principle attainable for all regions but will likely require further optimization of the primer sequences or the use of more than one primer combination to recover regions Sph4 and Sph5 throughout the genus. No relation between amplicon length and sequencing success is apparent. The shortest amplicon (Sph4, 109 bp) had the lowest success rate of all. This is additional indication that amplification success was biased by primer affinity. There is no indication for a taxonomic bias of primer affinity. Logistic regression revealed that the likelihood of sequencing success decreased, though again moderately, with specimen age for all amplicons. This negative relationship was significant (p<0.05) for four primer combinations and failed to achieve formal significance only by a narrow margin for amplicons Eois1 and Sph3 (Table 2). Total sequence length was negatively and significantly correlated with specimen age (r = −0.3850, p<0.001), though this relationship was not very strong (Fig. 2). This overall pattern is consistent with expectations and constitutes further testament to the authenticity of the obtained sequences. It is unlikely that unequal primer affinity would produce the observed pattern. Sequencing success for each specimen and primer combination is given in Table S1. All 91 specimens for which a sequence could be obtained yielded a sequence long enough to allow for unambiguous matching if a closely matching sequence was present in the data base. Even sequences as short as 120–140 bp can be sufficient for sequence matching [27], [36], provided that templates are sufficiently well known. Even if the cut-off for minimum sequence length was set to 200 bp one would have to eliminate only two type sequences from the dataset.

**Table 2:** Logistic regression: Results of univariate logistic regression analyses modelling the likelihood of sequencing success relative to specimen age for each region.

Amplicon	*β*	SE	t	p
Eois1	−0.028	0.015	−1.801	0.075
Eois2	−0.049	0.019	−2.573	**0.012**
Sph3	−0.027	0.015	−1.833	0.070
Sph4	−0.039	0.017	−2.257	**0.026**
Sph5	−0.034	0.014	−2.406	**0.018**
Eois6	−0.047	0.015	−3.065	**0.003**

Given are regression coefficients *β* for specimen age together with their respective *t*-statistics and p-values. Statistically significant p-values (0.05 confidence level) are indicated in bold. SE: Standard Error.

### Time and Cost

In total we performed 576 PCR reactions and 1,152 sequencing reactions. Processing cost per specimen was 29.20 Euros for lab reagents, consumables and sequencing, excluding cost for personnel and transport of specimens. This is equal to 4.7 times the cost to obtain a full COI barcode sequence from a recently collected specimen. Cost per specimen could have been reduced to 23.50 Euros (3.8× the cost of a fresh specimen) per specimen if unsuccessful PCR reactions would not have been sequenced. Still, more efficient protocols need to be developed to enable large scale processing of historical collection specimens. One step towards that goal may be achieved by sequencing only a subset of gene regions. If only the three most reliably amplifying regions (Eois1, Eois2, Sph3) were targeted, cost per specimen would be as low as 16.70 Euros (2.7× the cost of a fresh specimen). The resulting 359 bp long contiguous sequences would still be more than sufficient to make a robust sequence assignment. Compared to 6.20 Euros to obtain a full COI barcode sequence from a recently collected specimen the cost is still substantial in all three presented scenarios. Lab processing of all specimens from digestion of abdomens to performing sequencing reactions took 43 working hours. If processing is done in a streamlined way and not accounting for transport of samples between facilities but taking into account the time required for digestion, the entire dataset presented in this paper could have been generated within 9 days or less. This is less than the time required to prepare and properly document genitalia slides for the same amount of specimens. The cost for preparation and documentation of slides is comparable to the acquisition of barcodes from old specimens.

### Conclusion

The method described in this paper has proven to be unexpectedly effective in gaining sequences from historical type material. We believe that the use of insect abdomens is particularly advantageous for several reasons. 1. Abdomens contain orders of magnitude more DNA than legs. 2. Amplification of contaminant DNA is less likely when more endogenous DNA is present. 3. Abdomens are routinely removed for genitalic dissections, thus no additional damage is being inflicted to type specimens as compared to the sampling of legs. The quality of genitalia slides produced from proteinase-digested abdomens is equal to conventional maceration procedures using KOH. In practice the use of legs as DNA source for old specimens may be limited to larger specimens while abdomens will have to be used for small-sized specimens. We hope that other studies will investigate the utility of legs as a DNA source for old and valuable insect specimens. The use of multiplex PCR approaches may be required to make efficient use of DNA extracts from small tissue samples [37]. We encourage that the sequencing of barcodes from type specimens should become an indispensible part of every taxonomic revision or description of a new species whenever feasible. This will require that curators and collection managers make the types in their collection accessible for DNA sequencing. We hope that our study will encourage a more positive attitude towards DNA sequencing from type specimens in scientific collections around the world. In this study we were able to show that DNA sequencing of old types is possible with a sensible effort of cost and time. The sequences obtained here provided further evidence that the vast majority (88%) of *Eois* species at our study site in southern Ecuador are indeed undescribed. Approximately 40 preliminary species group assignments will have to be changed and three cases of suspected synonymy of valid described species could be detected. These results emphasize the exceedingly high value of genetic data extracted from historic type specimens to match biodiversity samples with stable zoological nomenclature, which again translates into more credibility of pertinent evolutionary, biogeographical and ecological studies. Barcode data generated in this study is currently being used in a step-wise taxonomic revision of *Eois* along with the description of newly discovered species.

## Supporting Information

Figure S1 **Neighbour-joining tree.** Neighbour-joining tree of sequences obtained in this study and 804 *Eois* reference barcode sequences. Specimens sequenced in this study are indicated in red.
(PDF)

Table S1 **List of material.** List of all sequenced *Eois* specimens with voucher codes, Genbank accession numbers, specimen age and obtained sequence length indicated. Sequencing success is indicated for each primer combination: 0: sequencing failed, 1: sequencing successful, overall success rates are indicated at the bottom of the table.
(XLSX)
