# Supplementary material for: DNA Barcode Sequencing from Old Type Specimens as a Tool in Taxonomy: A Case Study in the Diverse Genus Eois (Lepidoptera: Geometridae)
Source: PLoS One. 2012 Nov 21;7(11):e49710. doi: 10.1371/journal.pone.0049710 (PMC3504115; doi:10.1371/journal.pone.0049710)

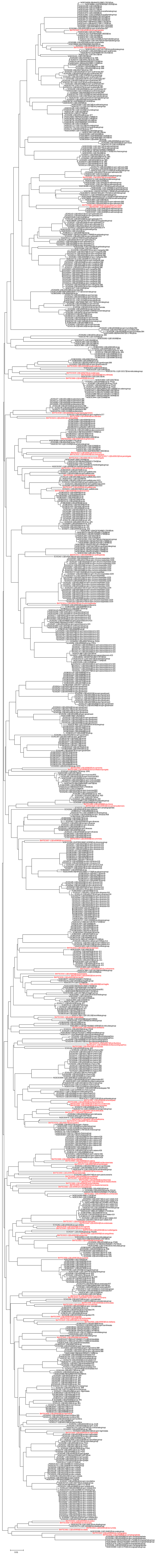

Supplement: Figure S1 — Neighbour-joining tree. Neighbour-joining tree of sequences obtained in this study and 804 Eois reference barcode sequences. Specimens sequenced in this study are indicated in red. (PDF) [file pone.0049710.s001.pdf]
